# Stress T1 mapping for the detection of obstructive coronary artery disease: a prospective diagnostic accuracy study

**DOI:** 10.1016/j.jocmr.2026.102701

**Published:** 2026-01-29

**Authors:** Simran Shergill, Mohamed Elshibly, Anju Velvet, Aida Moafi, Rachel England, Kelly S. Parke, Joanne V. Wormleighton, David Adlam, Sandeep S. Hothi, Peter Kellman, Alasdair McIntosh, Alex McConnachie, Andrew Ladwiniec, Gerry P. McCann, J. Ranjit Arnold

**Affiliations:** a Department of Cardiovascular Sciences, University of Leicester, the National Institute for Health and Care Research Leicester Biomedical Research Centre and British Heart Foundation Centre of Research Excellence, Glenfield Hospital, Leicester, UK; b Heart and Lung Centre, Royal Wolverhampton NHS Trust, Wolverhampton, UK; c Institute of Cardiovascular Sciences, University of Birmingham, Birmingham, UK; d National Heart, Lung, and Blood Institute, National Institutes of Health, Department of Health and Human Services, Bethesda, Maryland, USA; e Robertson Centre for Biostatistics, School of Health and Wellbeing, University of Glasgow, Glasgow, UK; f Department of Cardiology, University Hospitals of Leicester NHS Trust, Leicester, UK

**Keywords:** Myocardial ischemia, Contrast-free, MOLLI T1 mapping, Fractional flow reserve, Stress-perfusion CMR

## Abstract

**Background:**

In the assessment of patients with suspected coronary artery disease (CAD), the diagnostic role of stress-perfusion cardiovascular magnetic resonance (CMR) is well established. However, its reliance on gadolinium-based contrast agents may restrict its application in certain populations. T1 mapping during vasodilatory stress has been proposed as a contrast-free alternative for detecting CAD. This study sought to compare the diagnostic accuracy of adenosine-stress T1 reactivity (ΔT1) with that of stress-perfusion CMR for identifying hemodynamically significant CAD.

**Methods:**

Patients with suspected angina referred for diagnostic invasive coronary angiography underwent 3-Tesla CMR consisting of the following: (1) T1 mapping at rest and following intravenous adenosine using a modified Look-Locker inversion recovery sequence, (2) stress and rest perfusion, and (3) late-gadolinium enhancement. Significant CAD was defined invasively as fractional flow reserve ≤0.80 in epicardial vessels ≥2 mm diameter (or quantitative flow ratio ≤0.80 if unavailable). A ΔT1 vessel threshold (% increase in T1 from rest to stress) was derived from receiver operating characteristic analysis, using invasive coronary angiography as the reference standard. Stress-perfusion CMR was assessed qualitatively with CAD determined by the presence of ischemia and/or infarction, (A) per-vessel (as determined by two independent readers) and (B) per-patient (following consensus read).

**Results:**

Of 121 prospectively recruited patients, 115 had paired T1 mapping and coronary angiography data (mean age 66 ± 9 years, 72% [83/115] male, CAD prevalence 51% [59/115]). ΔT1 demonstrated poor diagnostic performance for detecting significant CAD (area under the curve 0.59 [95% CI: 0.52, 0.65], p = 0.011), with an optimal vessel threshold ≤4.36% giving accuracy 54.9%, sensitivity 68.3%, and specificity 49.2%. Stress-perfusion CMR demonstrated superior diagnostic accuracy compared to ΔT1: (A) per-vessel (for the two independent reads, +26.2% [19.4%, 32.6%] and +26.7% [19.9%, 33.3%], both p<0.001) and (B) per-patient (for consensus read, +21.7% [10.2%, 32.6%], p<0.001).

**Conclusion:**

In patients with suspected angina, ΔT1 demonstrates limited diagnostic accuracy for the detection of obstructive CAD. Future efforts should be directed toward alternative contrast-free methods for the reliable detection of CAD in this population.

## Graphical abstract

**Figure:**
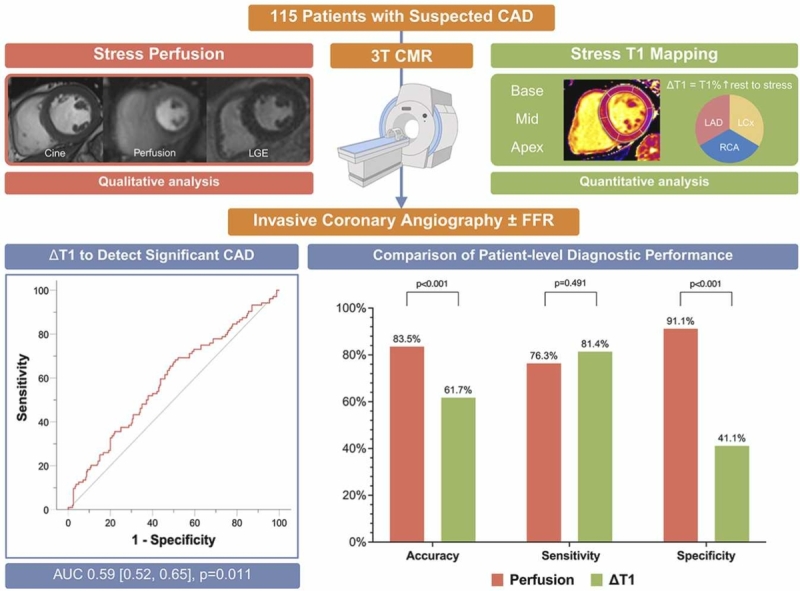
*AUC* area under the curve, *CAD* coronary artery disease, *CMR* cardiovascular magnetic resonance, *FFR* fractional flow reserve, *LAD* left anterior descending artery, *LCx* left circumflex artery, *LGE* late-gadolinium enhancement, *RCA* right coronary artery, *T* tesla.

## 1 Introduction

Stress-perfusion cardiovascular magnetic resonance (CMR) is a well-established, non-invasive diagnostic tool for evaluating patients with known or suspected coronary artery disease (CAD) [1], [2]. It serves as an effective “gatekeeper” for invasive testing, reducing unnecessary revascularization and providing robust prognostication [3], [4], [5]. Stress-perfusion CMR provides high diagnostic accuracy for the detection of myocardial ischemia [6], but is reliant on gadolinium-based contrast, which can prolong examination times and limit accessibility in patients with contraindications [7], [8], [9], [10]. These practical considerations highlight the need to explore alternative, contrast-free methods to detect ischemia.

Native T1 mapping is a contrast-free parametric technique that allows the pixel-wise quantification of myocardial tissue characteristics by measuring the longitudinal relaxation time constant [11]. T1 relaxation times are sensitive to changes in intramyocardial free water content across the intracellular, extracellular, and intravascular compartments, making T1 mapping a powerful tool for detecting diffuse myocardial abnormalities such as edema, fibrosis, and infiltration [12]. Applied under vasodilatory stress, T1 mapping may also detect changes in myocardial blood volume (MBV), which may serve as a marker of ischemia [13], [14], [15], [16].

Although T1 reactivity (ΔT1) has emerged as a potential marker of ischemia [17], [18], its diagnostic accuracy for the detection of obstructive CAD remains undefined, with a lack of appropriately powered, prospective studies using invasive reference standards. Therefore, we conducted a prospective diagnostic accuracy study to evaluate the diagnostic performance of ΔT1 against the invasive reference standard of fractional flow reserve (FFR), and using standard adenosine-stress perfusion CMR as the comparator.

## 2 Methods

### 2.1 Study design

One-hundred and twenty-one adult patients with *de novo* suspected angina referred for clinically indicated diagnostic invasive coronary angiography (ICA) were prospectively recruited from a single tertiary cardiac centre (Glenfield Hospital, Leicester, United Kingdom) between September 2021 and April 2024. The design of this pre-specified analysis has been described in detail elsewhere (NCT04761991) [19]. Participants underwent research CMR prior to ICA, which was performed blinded to imaging results. Exclusion criteria were recent myocardial infarction (≤6 months), unstable angina, previous revascularization, contraindications to adenosine (second/third-degree atrioventricular block, severe chronic obstructive pulmonary disease, moderate-severe asthma), severe renal dysfunction (estimated glomerular filtration rate <30 mL/min/1.73 m^2^), severe claustrophobia and absolute contraindications to CMR (non-conditional cardiac implantable electronic device, pregnancy, ferromagnetic implants/foreign bodies). Ethical approval was granted by the United Kingdom National Research Ethics Service (REC reference 19/EM/0295). The study was conducted in accordance with the Declaration of Helsinki and all participants gave written informed consent prior to participation.

### 2.2 Cardiovascular magnetic resonance

CMR was conducted at 3-Tesla (Vida or Skyra, Siemens Healthineers, Erlangen, Germany) with electrocardiographic (ECG) gating and an 18-channel phased-array cardiac receiver coil. Participants were advised to abstain from caffeine-containing products for at least 12 h prior to vasodilator stress, but routine anti-anginal medications were continued. Hyperemia was induced with adenosine at a rate of 140 μg/kg/min for 3–5 min. Subjects were monitored for symptoms throughout the infusion, with dose escalations at two-minute intervals to 170–210 μg/kg/min if there was an insufficient symptomatic and/or hemodynamic response (heart rate increase of ≥10 beats per minute) [20].

### 2.3 CMR protocol

**Fig. 1:**
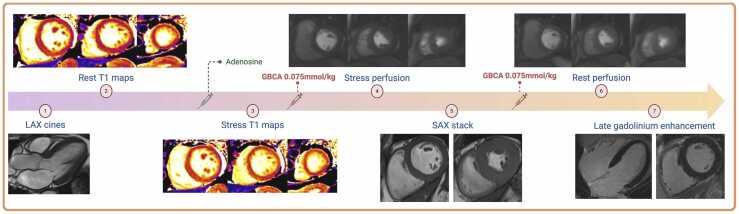
CMR protocol. *GBCA* gadolinium-based contrast agent, *LAX* long-axis, *SAX* short-axis

The CMR protocol has previously been described [19] **(**Fig. 1**)**. In brief, functional cine imaging was performed in the three long-axis planes (4-, 2-, 3-chamber) and, after stress-perfusion, a contiguous short-axis stack to cover the ventricles using a breath-hold, steady-state free precession pulse sequence. Native T1 mapping was performed at rest and peak adenosine stress at the basal, mid-ventricular and apical levels of the left ventricle using an end-expiratory breath-hold, ECG-gated, modified Look-Locker inversion recovery (MOLLI) sequence (Siemens MyoMaps, Erlangen, Germany) with a 5(3)3 acquisition scheme, with inline motion-corrected reconstructions using a balanced steady-state free precession single-shot readout in end-diastole (typical sequence parameters: TE 1.06 ms, TR 274.8 ms, slice thickness 8 mm, matrix 256 × 144, FOV 360 mm, FOV phase 85.2%, pixel size 1.4 × 1.9 mm^2^, flip angle 35°) [12], [21]. A dual-sequence T1-weighted saturation-recovery gradient echo sequence was performed immediately after T1 mapping at peak vasodilator stress and at rest, with matched slice prescription to T1 maps and injection of 0.075 mmol/kg of gadoterate meglumine (Dotarem, Guerbet, France or Clariscan, GE HealthCare, Chalfont Saint Giles, UK) for each perfusion scan at 4 mL/s, followed by a 20 mL 0.9% saline bolus [22]. Late-gadolinium enhancement (LGE) imaging was acquired using a breath-hold, T1-weighted segmented inversion recovery gradient echo sequence in the same long and short-axis slice prescriptions as the cine imaging, with the optimal inversion time determined from a Look-Locker sequence.

### 2.4 Image analysis

CMR images were analyzed offline, blinded to all participant and angiographic details using certified software (CVI42, v. 6.1.2; Circle Cardiovascular Imaging, Calgary, Alberta, Canada). Image anonymization was performed by an independent administrator, and unblinding codes held by staff independent from the study investigators. Volumetric, perfusion and LGE analysis has been previously described [19]. For stress-perfusion CMR, qualitative interpretation of cine, perfusion and LGE images (with exclusion of T1 maps) was performed by two experienced level 3 CMR accredited observers acting in consensus following two independent reads, using the 16-segment American Heart Association model, further subdivided into subepicardial and subendocardial layers (32-segment model) for perfusion and LGE assessment, with segments ascribed to a coronary territory according to standard criteria [23]. For qualitative LGE assessment, scar patterns were graded at a segmental level as follows: 0 = normal, 1 = subendocardial, 2 = transmural, 3 = non-ischemic, 4 = insertion point fibrosis. For visual qualitative perfusion assessment, standard segmental scoring was applied as follows: 0 = normal, 1 = subendocardial defect, 2 = transmural defect. Ischemia was defined as a stress-induced defect in two adjacent segments of a 32-segment model, more extensive than either resting perfusion defect or infarction on matched LGE imaging [24]. Significant CAD was determined on per-vessel (by two independent readers) and per-patient (following consensus read) levels, defined by the presence of ischemia and/or infarction.

Inline, motion-corrected T1 maps were analyzed by a single observer, blinded to perfusion, LGE, and ICA data. T1 map quality was graded on a 4-point scale as follows: 3 = excellent (no artifact and fully diagnostic), 2 = good (minor artifact but fully diagnostic), 1 = moderate (noticeable artifact with some diagnostic limitation), and 0 = unanalyzable (severe artifact rendering the segment non-diagnostic). Segments with artifacts were excluded from analysis (e.g., susceptibility, wrap, off resonance, or motion-related artifact). Endocardial and epicardial borders for rest and stress T1 maps were automatically defined using the built-in automated contouring tool, with manual adjustments for clear and obvious errors. To minimize the risk of blood pool inclusion or partial volume effects, a 10% offset was applied to each border. Mean myocardial segmental T1 (ms) was recorded using the 16-segment American Heart Association model, with segments ascribed a coronary territory according to standard criteria and adjusted for coronary dominance as identified during ICA [23]. Previous internal validation has shown no systematic differences in T1 values between the scanners used in this study (Supplementary Table 1). For comparisons of T1 values, segments were averaged either by coronary territory or globally. For the primary analysis, rest and stress T1 values were averaged at the coronary territory level, and ΔT1 was calculated as previously described [25]:

$$\Delta T1\%=\left(\frac{\mathrm{Stress}\;T1-\mathrm{Rest}\;T1}{\mathrm{Rest}\;T1}\right)\times 100$$

### 2.5 Invasive coronary angiography protocol and analysis

All participants had been referred for clinically indicated ICA, which was performed according to standard clinical protocols by interventional cardiologists blinded to imaging findings. FFR was performed in epicardial vessels with visually determined stenosis of 40%–90% using an intracoronary pressure wire (PressureWire X, Abbott Vascular, Illinois) and 6-French guide catheter. Hyperemia was induced with intravenous adenosine and FFR was calculated as the ratio of mean distal coronary pressure to mean aortic pressure, adjusted for pressure drift [26]. Significant CAD was defined as FFR ≤0.80 in an epicardial vessel ≥2 mm diameter. In vessels deemed not safe to perform FFR (subtotal or complete occlusions), the vessel was assumed to have FFR 0.50. In remaining vessels in which FFR had not been determined and with visually determined ≥25% (mild) stenosis, quantitative flow ratio (QFR) computation was performed offline by an independent observer blinded to imaging and clinical details, using QFR v2.2 software (Medis Medical Imaging, Leiden, the Netherlands) with methods as previously described [19]. QFR ≤0.80 was considered significant [2].

### 2.6 Statistical analysis

Statistical analyses were overseen by an independent Clinical Trials Unit at the University of Glasgow. Continuous data are expressed as mean ± standard deviation if normally distributed or median [Q1–Q3] if otherwise. Categorical data are presented as counts and percentages (%). The primary analysis compared the diagnostic accuracy of ΔT1 to that of stress-perfusion CMR for the detection of hemodynamically significant CAD at the vessel level. Secondary analyses evaluated as follows: (1) patient and vessel level diagnostic performance of ΔT1 and stress T1 (accuracy, sensitivity, specificity), (2) the ability of T1 mapping to distinguish CAD from non-CAD territories, and (3) whether CAD was an independent determinant of segmental T1 response. Independent or paired sample t-tests compared group differences where appropriate. Receiver operating characteristic analyses (ROC) assessed the diagnostic performance of T1 mapping, with reporting of area under the curve (AUC [95% confidence interval (CI)]) and were compared using the DeLong method. The Youden index was used to derive optimal thresholds at the vessel level. For patient-level analyses, vessel-specific thresholds were applied, with a patient considered positive if at least one territory fell below the defined threshold. Two-sided 95% CI for differences in accuracy, sensitivity, and specificity were obtained using the Newcombe-Wilson method and differences in proportions were compared using McNemar’s test. A linear mixed-effects analysis was carried out to account for the within-patient correlation of segmental T1 data. A p-value <0.05 was considered statistically significant. Statistical analysis was performed using the Statistical Package for Social Sciences version 29.0 (IBM Corp., Armonk, New York, USA) and R Foundation for Statistical Computing version 4.4.3 (Vienna, Austria).

### 2.7 Sample size

For sample size calculation, we assumed a diagnostic accuracy of 85% for stress-perfusion CMR and 75% for ΔT1. To demonstrate superiority of stress-perfusion CMR at the per-vessel level, 337 complete vessel pairs were required for 90% power (α significance 5%), assuming both protocols disagree no more than 20% of the time. If ΔT1 achieved a diagnostic accuracy of 80%, assuming no more than 10% discordant pairs, this sample size would also provide 80% power to demonstrate superiority of stress-perfusion CMR at the vessel level. To allow for missing data in up to 10%, the target sample size was 124 participants.

## 3 Results

### 3.1 Subject characteristics

One-hundred and fifteen patients were included in the final analyses (mean age 66 ± 9 years, 72% [83/115] male). Five patients did not have T1 maps at peak adenosine-stress due to poor breath-holding capacity and in one patient, both the rest and stress T1 maps were unanalyzable. Baseline characteristics are presented in Table 1. The median interval between CMR and ICA was 26 [12–44] days. From angiographic analysis, significant CAD prevalence was 51% (27 [24%] with single-, 19 [17%] with double-, and 13 [11%] with triple-vessel disease).

**Table 1:** Baseline characteristics

Participants with suspected angina referred for ICA, n = 115		
*Demographics*		
Age, years	66±9	
Male	83 (72%)	
Body mass index, kg/m^2^	29.5±4.8	
*Cardiovascular risk factors*		
Current or ex-smoker	59 (51%)	
Hypertension	72 (63%)	
Type II diabetes	16 (14%)	
Hypercholesterolemia	52 (45%)	
Family history of premature CAD	50 (44%)	
Previous myocardial infarction	2 (2%)	
*Medications*		
Aspirin	88 (77%)	
Statin	103 (90%)	
ACEi/ARB	44 (38%)	
Beta blocker	72 (63%)	
Calcium channel blocker	41 (36%)	
Nitrate	46 (40%)	
*Left ventricular function*		
Ejection fraction, %	61.0±8.9	
End-diastolic volume index, mL/m^2^	75.7±14.7	
End-systolic volume index, mL/m^2^	29.8±10.0	
Mass index, g/m^2^	59.6±11.8	
Hemodynamics*	*Baseline*	*Peak stress*
HR, bpm	62±10	82±12
SBP, mmHg	139±20	138±20
DBP, mmHg	78±10	77±12
*LGE*		
Infarction	17 (15%)	
Total infarcted segments	42	
Enhanced mass, g†	6.03 [2.97, 10.12]	
Non-ischemic focal fibrosis	15 (13%)	
*Invasive coronary angiography*		
Functionally significant stenosis	59 (51%)	
Single-vessel disease	27 (24%)	
Double-vessel disease	19 (17%)	
Triple-vessel disease	13 (11%)	
Complete or subtotal occlusion	22 (19%)	

Data presented as mean ± SD, median [Q1, Q3] or counts (%).

*ACEi/ARB* angiotensin-converting enzyme inhibitor/angiotensin receptor blocker, *BPM* beats per minute, *CAD* coronary artery disease, *DBP* diastolic blood pressure, *HR* heart rate, *ICA* invasive coronary angiography, *LGE* late-gadolinium enhancement, *SBP* systolic blood pressure

* Blood pressure not available: n = 1.

† In those with infarction.

### 3.2 Baseline hemodynamics and response to adenosine infusion

Table 1 summarizes the hemodynamic response to adenosine. Thirty-eight percent (44/115) of participants required an adenosine dose increase, with 13% (15/115) requiring the maximum dose of 210 μg/kg/min. At peak adenosine stress, 92% (106/115) of participants achieved a satisfactory heart rate response ≥10 beats per minute, with a mean increase of 19 ± 8 bpm (p<0.001).

### 3.3 Image quality

Image quality of rest and stress T1 maps was rated as “excellent” or “good” for the majority of cases (Supplementary Table 2). At rest, 33 segments (1.8%) were excluded due to artifact, compared with 174 segments (9.5%) at stress. As a result, only one rest T1 territory was not available for analysis, providing 344 complete vessel pairs for the primary analysis.

### 3.4 Native T1 response to adenosine infusion

**Fig. 2:**
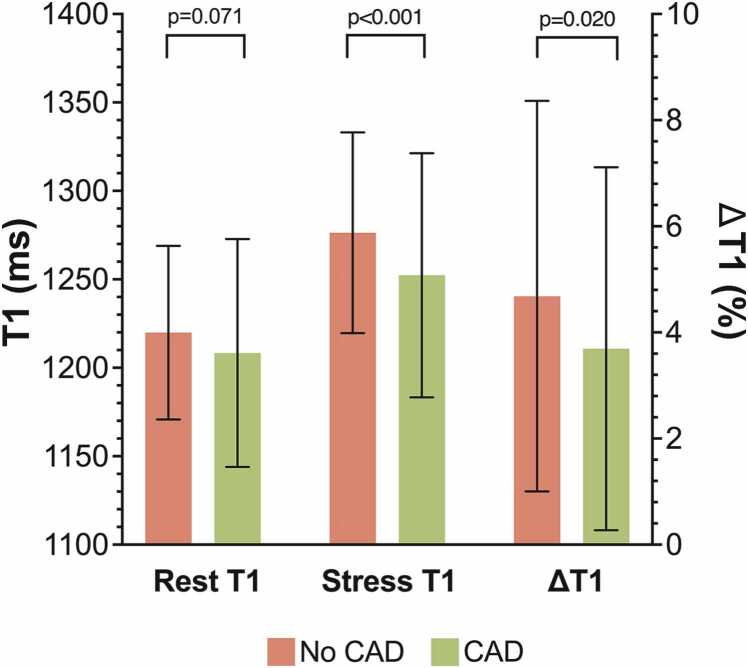
Native T1 and ΔT1 response stratified by the presence of CAD Graphical data presented as mean ± SD. *CAD* coronary artery disease, *SD* standard deviation

At rest, the global native T1 was 1217 ± 49ms, increasing to 1270 ± 55ms at peak adenosine stress (p<0.001) (Supplementary Table 3 and see Supplementary Table 4 for sex-specific T1 responses). A significant increase in T1 was observed in myocardial territories both with and without CAD (Supplementary Table 3). At the vessel level, rest T1 values were similar between territories with and without CAD. Stress T1 values were marginally but significantly lower in vessels with CAD than in those without CAD, with corresponding attenuation of ΔT1 (3.69 ± 3.42% vs. 4.68 ± 3.68%, mean difference −0.99% [−1.82%, −0.16%]; p = 0.020) **(**Table 2**,** Fig. 2**)**.

**Table 2:** Comparison of native T1 and ΔT1 response stratified by the presence of CAD

Per vessel, n = 345	CAD, n = 104	No CAD, n = 241	Mean difference [95% CI]	p value
Rest T1†, ms	1208.3±64.4	1219.9±49.1	-11.5 [−24.0, 1.0]	0.071
Stress T1, ms	1252.4±69.0	1276.3±56.8	-24.0 [−38.0, −10.0]	<0.001
ΔT1, %	3.69±3.42	4.68±3.68	-0.99 [−1.82, −0.16]	0.020

Per patient, n = 115	CAD, n = 59	No CAD, n = 56	Mean difference [95% CI]	p value
Rest T1, ms	1209.1±56.9	1224.8±37.2	-15.7 [−33.6, 2.1]	0.084
Stress T1, ms	1254.7±57.3	1285.9±48.4	-31.2 [−50.8, −11.5]	0.002
ΔT1, %	3.82±2.84	5.01±3.42	-1.20 [−2.36, −0.04]	0.043

*CAD* coronary artery disease.

Data presented as mean ± SD and mean difference [95% confidence interval].

† Rest T1 left circumflex territory excluded due to artifact: n=1

### 3.5 Diagnostic performance of T1 mapping to detect significant CAD

#### 3.5.1 Per vessel analysis

**Fig. 3:**
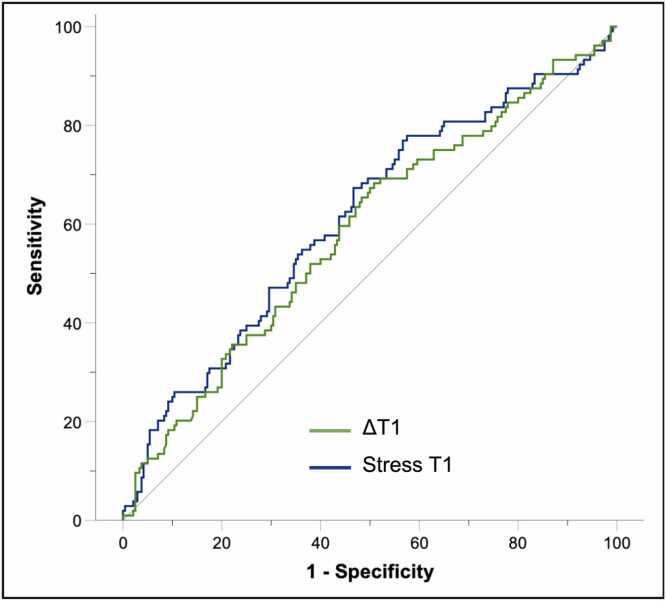
Receiver operating characteristic analyses demonstrating the poor diagnostic performance of ΔT1 (area under the curve 0.59 [0.52, 0.65], p = 0.011) and stress T1 (0.61 [0.55, 0.68], p = 0.001) to detect significant coronary artery disease at the vessel level, using invasive coronary angiography as the reference standard

**Fig. 4:**
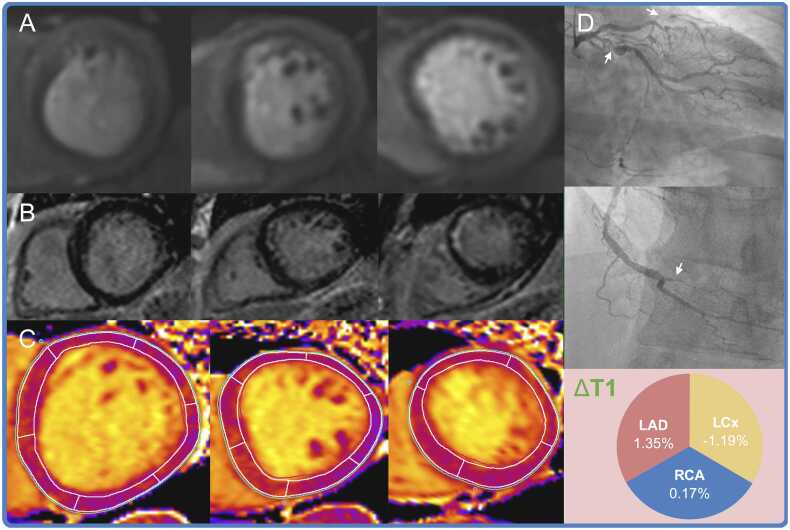
Concordant case example. Stress-perfusion (Panel A) demonstrates a heterogeneous circumferential perfusion defect across all three ventricular levels, exceeding the subendocardial scar in the septum on late-gadolinium enhancement (Panel B). T1 mapping during adenosine-stress (Panel C) shows a blunted ΔT1 response in all three coronary territories (threshold ≤4.36%). Both the stress-perfusion and T1 mapping were consistent with significant three-vessel coronary artery disease. This was confirmed on invasive coronary angiography (Panel D), with complete total occlusions of the mid left anterior descending and distal right coronary arteries and a severe proximal left circumflex artery lesion (arrowed). *LAD* left anterior descending artery, *LCx* left circumflex artery, *RCA* right coronary artery

**Fig. 5:**
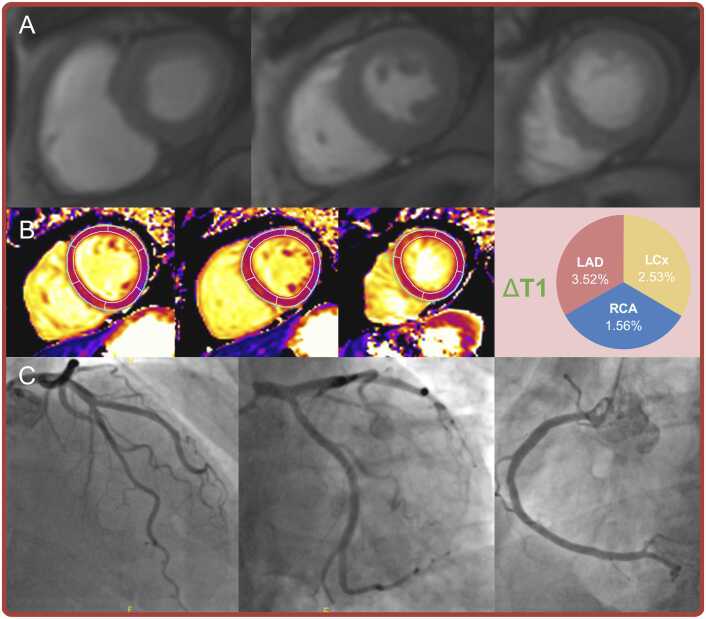
Discordant case example. No discernible inducible ischemia on stress-perfusion (Panel A) with invasive coronary angiography confirming the presence of normal epicardial coronary arteries (Panel C). However, adenosine-stress T1 mapping demonstrates a blunted ΔT1 response in all three coronary territories (threshold ≤4.36%), suggesting the presence of three-vessel epicardial coronary artery disease. *LAD* left anterior descending artery, *LCx* left circumflex artery, *RCA* right coronary artery

In the primary analysis, the diagnostic performance of ΔT1 to detect significant CAD at the vessel level was poor (AUC 0.59 [0.52, 0.65], p = 0.011), with an optimal threshold ≤4.36% yielding accuracy 54.9%, sensitivity 68.3% and specificity 49.2% **(**Table 3**,** Fig. 3**)**. Stress-perfusion CMR demonstrated superior diagnostic accuracy compared to ΔT1 for reader 1 (81.1% vs. 54.9%, mean difference +26.2% [19.4%, 32.6%], p<0.001) and for reader 2 (81.7% vs. 54.9%, mean difference +26.7% [19.9%, 33.3%], p<0.001; Table 4**,** Fig. 4, Fig. 5 for case examples). Sensitivity did not differ significantly between stress-perfusion CMR and ΔT1 for either reader; however, stress-perfusion demonstrated dramatically higher specificity than ΔT1 for both readers **(**Table 4**)**.

**Table 3:** Diagnostic performance of ΔT1, stress, and rest T1 for the detection of significant CAD at the vessel level

n = 345	AUC [95% CI]	p value	Threshold	Accuracy	Sensitivity	Specificity
ΔT1, %	0.59 [0.52, 0.65]	0.011	≤4.36	54.9%	68.3%	49.2%
Stress T1, ms	0.61 [0.55, 0.68]	0.001	≤1273.9	57.4%	67.3%	53.1%
Rest T1†, ms	0.55 [0.49, 0.62]	0.106	≤1232.7	51.2%	68.3%	43.7%

*AUC* area under the curve, *CAD* coronary artery disease, *CI* confidence interval*.*

Significant CAD defined by invasive FFR ≤0.80 in epicardial vessels ≥2 mm diameter, or QFR ≤0.80 if FFR not performed

† Rest T1 LCx territory excluded due to artifact: n = 1

**Table 4:** Comparison of the diagnostic performance between ΔT1 with stress-perfusion CMR to detect significant CAD, using invasive coronary angiography as the reference standard

*Per vessel, n = 344*	Stress-perfusion CMR	ΔT1	Mean difference [95% CI]	p value
*Reader 1*				
Accuracy	81.1% [76.5%, 85.5%]	54.9% [50.0%, 59.9%]	26.2% [19.4%, 32.6%]	<0.001
Sensitivity	61.5% [51.9%, 71.2%]	68.3% [59.6%, 76.9%]	-6.7% [−19.5%, 6.4%]	0.317
Specificity	89.6% [85.4%, 93.3%]	49.2% [42.9%, 55.4%]	40.4% [33.3%, 47.0%]	<0.001
	
*Reader 2*				
Accuracy	81.7% [77.0%, 85.8%]	54.9% [50.0%, 59.9%]	26.7% [19.9%, 33.3%]	<0.001
Sensitivity	57.7% [48.1%, 66.3%]	68.3% [59.6%, 76.9%]	-10.6% [−23.7%, 3.0%]	0.131
Specificity	92.1% [88.3%, 95.4%]	49.2% [42.9%, 55.4%]	42.9% [35.9%, 49.4%]	<0.001

*Per patient, n = 115*	Stress-perfusion CMR	ΔT1	Mean difference [95% CI]	p value
*Consensus*				
Accuracy	83.5% [76.5%, 89.6%]	61.7% [52.2%, 71.3%]	21.7% [10.2%, 32.6%]	<0.001
Sensitivity	76.3% [64.4%, 86.4%]	81.4% [71.2%, 91.5%]	-5.1% [−19.4%, 9.4%]	0.491
Specificity	91.1% [83.9%, 98.2%]	41.1% [28.6%, 53.6%]	50.0% [33.9%, 62.6%]	<0.001

Proportions expressed as percentage [95% confidence interval].

Two-sided p value for difference.

*CAD* coronary artery disease, *CI* confidence interval, *CMR* cardiovascular magnetic resonance

Significant CAD defined by invasive FFR ≤0.80 in epicardial vessels ≥2 mm diameter, or QFR ≤0.80 if FFR not performed

In the secondary analysis evaluating stress T1, the diagnostic performance for detecting significant CAD was also poor (AUC 0.61 [0.55, 0.68], p = 0.001; Table 3), similar to that of ΔT1 (difference in areas 0.026 [−0.046, 0.098], p = 0.475) but significantly inferior to that of stress perfusion CMR (Supplementary Table 5).

### 3.6 Per patient analysis

When comparing patient-level diagnostic performance, stress-perfusion CMR demonstrated superior diagnostic accuracy and specificity, while sensitivity was comparable to both ΔT1 (Table 4) and stress T1 (Supplementary Table 5).

### 3.7 Segmental analysis

Segmental-based methods (lowest, second lowest, and mean of two lowest adjacent segments per coronary territory) and analysis of dispersion (standard deviation of segments within a coronary territory) using either ΔT1 or stress T1 demonstrated comparable diagnostic performance and did not significantly outperform territory-based approaches (Supplementary Table 6).

In a segmental level, linear mixed-effects model, CAD remained an independent predictor of ΔT1 (β −0.81, p = 0.016) and stress T1 (β −10.38, p = 0.005) following adjustments for age, sex, body mass index, cardiovascular risk factors, beta blocker use, left ventricular ejection fraction, heart rate variability during adenosine stress and presence of segmental infarction on LGE **(**Table 5**)**. Furthermore, male sex and presence of hypertension were independently associated with a blunted ΔT1 response **(**Table 5**).**

**Table 5:** Adjusted associations between clinical variables and segmental T1 responses based on a linear mixed-effects model

ΔT1, n = 1655	Estimate (β)	p value	95% CI
Age	−0.04	0.198	[−0.11, 0.02]
Sex*	−1.53	0.017	[−2.78, −0.27]
Body mass index	0.11	0.096	[−0.02, 0.24]
Hypertension	−1.33	0.040	[−2.59, −0.06]
Type II diabetes	1.34	0.108	[−0.30, 2.99]
Current or ex-smoker	0.99	0.105	[−0.21, 2.20]
Beta blockade	−0.63	0.304	[−1.85, 0.58]
LVEF	0.01	0.832	[−0.06, 0.07]
CAD	−0.81	0.016	[−1.46, −0.15]
Infarction	−0.15	0.851	[−1.68, 1.38]
Peak stress HR	0.02	0.555	[−0.05, 0.09]
Baseline HR	−0.16	<0.001	[−0.24, −0.07]

Stress T1, n = 1666	Estimate (β)	p value	95% CI
Age	−0.67	0.095	[−1.47, 0.12]
Sex*	−25.24	<0.001	[−39.94, −10.54]
Body mass index	1.29	0.093	[−0.22, 2.80]
Hypertension	−14.05	0.063	[−28.87, 0.78]
Type II diabetes	16.81	0.087	[−2.50, 36.12]
Current or ex-smoker	10.22	0.155	[−3.93, 24.36]
Beta blockade	−5.72	0.429	[−20.02, 8.57]
LVEF	0.03	0.936	[−0.70, 0.76]
CAD	−10.38	0.005	[−17.69, −3.08]
Infarction	8.10	0.349	[−8.87, 25.08]
Rest T1	0.65	<0.001	[0.60, 0.70]
Peak stress HR	−0.32	0.443	[−1.15, 0.51]
Baseline HR	−1.03	0.045	[−2.03, −0.02]

β coefficients represent adjusted mean differences for categorical variables and a one-unit increase in age, body mass index, HR, LVEF or rest T1. *CAD* coronary artery disease, *CI* confidence interval, *HR* heart rate, *LVEF* left ventricular ejection fraction

* Females as reference.

## 4 Discussion

In this prospective, powered and robustly conducted clinical study, we demonstrate that the diagnostic performance of stress and ΔT1 to detect hemodynamically significant CAD is poor and considerably worse than stress-perfusion CMR. This study represents the largest prospective evaluation of stress T1 mapping in patients with suspected CAD, with ICA available in all participants and qualitative stress-perfusion CMR as a comparator. These findings provide definitive negative evidence, indicating the limited clinical utility of stress T1 mapping in CAD detection and supporting the redirection of future research priorities towards more promising CMR strategies. Previous studies are limited by smaller sample sizes and the use of heterogeneous or exclusively non-invasive reference standards. By contrast, our prospective, blinded methodology with an invasive reference standard provides rigorous assessment of the technique, demonstrating conclusively the limited diagnostic utility of vasodilatory stress T1 mapping for the detection of significant CAD. Although there were modest differences in stress T1 and ΔT1 between coronary territories with and without CAD, our findings confirm that T1 mapping under vasodilatory stress does not provide sufficient diagnostic performance for the detection of CAD. Future research should be directed towards alternative methods for the reliable detection of obstructive CAD in this patient population.

Stress-induced increases in myocardial T1 are thought to reflect changes in MBV during hyperemia [16]. This concept was examined in a study of 41 healthy subjects, which demonstrated that native T1, T2, extracellular volume fraction and myocardial perfusion all increased during adenosine stress. Notably, both myocardial perfusion and extracellular volume fraction independently predicted the T1 response, suggesting that alterations in T1 during vasodilatory stress reflect increased MBV [27].

Consistent with our findings, previous clinical studies have demonstrated an attenuated ΔT1 response in patients with ischemic or infarcted myocardium, suggesting that ΔT1 may serve as a potential diagnostic tool in clinical practice [17], [18], [28], [29], [30]. In the presence of significant epicardial stenoses, reduced capillary reserve fails to further augment coronary blood flow during hyperemia, leading to a blunted MBV response and consequent attenuated stress T1 response [18].

To date, studies assessing the diagnostic performance of ΔT1 have been limited. A previous study of 43 patients with suspected CAD evaluated the diagnostic accuracy of ΔT1 using a shortened MOLLI sequence at a single mid-ventricular slice [31]. Absolute myocardial blood flow (MBF) as measured by adenosine-stress positron emission tomography served as the reference standard, with impaired perfusion defined by stress MBF ≤2.3 mL/min/g and myocardial perfusion reserve (MPR) ≤2.5. The authors found that ΔT1 was lower in territories with impaired MBF but not MPR. Furthermore, the diagnostic accuracy of ΔT1 for detecting ischemia was poor, with an AUC of [0.57, 0.75] using stress MBF as the reference standard and 0.62 [0.53, 0.71] with MPR.

A previous study utilizing qualitative stress-perfusion CMR as the reference standard assessed the utility of ΔT1 using a MOLLI sequence in 91 patients with known or suspected CAD [32]. Despite a significant difference in ΔT1 between segments with and without ischemia, the diagnostic performance of ΔT1 was again poor (AUC 0.68 [0.61, 0.74]) with accuracy only improving when readers were unblinded to the perfusion images, and focal regions of interest were selected for T1 measurements (AUC 0.85 [0.78, 0.91]). However, this approach diminishes clinical utility, as it is reliant on the availability of additional perfusion imaging, which necessitates contrast administration and prolongs scan duration. In an extension of this work to 184 patients, the diagnostic performance of ΔT1 did not improve (AUC 0.60 [0.58, 0.62]) [33]. However, the high prevalence of scar (n = 76) in this population may have altered resting T1 times, potentially confounding the stress T1 response, and additionally, the lack of inline motion-corrected sequences may have further compromised performance.

A retrospective study of 51 patients with suspected CAD (previous revascularization in 31% and infarction in 26%) utilized 3-Tesla CMR with MOLLI T1 mapping and quantitative perfusion [34]. In line with our findings, stress T1, and consequently ΔT1 were significantly lower in ischemic territories. Similarly, the diagnostic performance of ΔT1 to detect ischemia was again poor (AUC 0.66 [0.56, 0.75]), using a previously defined MPR threshold <1.96 [35]. In the limited vessels with ICA available (n=60), both stress and ΔT1 failed to differentiate territories with and without CAD. However, this study is limited by its small sample size, incomplete availability of ICA data in all patients, the absence of comparison with qualitative stress-perfusion CMR and the use of a pre-defined quantitative perfusion threshold. Nonetheless, this study does further highlight the limited diagnostic utility of stress T1 approaches. By contrast, our prospective study benefits from a larger sample size, with ICA available in all patients (with 344 vessels evaluated as opposed to 60), and the inclusion of qualitative stress-perfusion CMR as a comparator.

Although CAD was an independent determinant of the segmental ΔT1 response, the observed differences did not translate into clinically meaningful discrimination, as reflected by the poor AUC values. The limited diagnostic accuracy may stem in part from the relatively small overall difference in T1 values between CAD and non-CAD territories with considerable overlap. Since arteriolar and venular volumes comprise only 10% of MBV, their dilation during stress produces only a modest increase in overall MBV, typically within a limited range of 3 to 5%, which is markedly lower than the ∼300–400% increase in MBF observed during vasodilator stress [36]. Consequently, the absolute changes in myocardial T1 times between diseased and non-diseased territories, although statistically significant, remain relatively small and not clinically meaningful, thereby limiting the diagnostic utility of stress T1 mapping [31].

## 5 Limitations

Despite the prospective, blinded, and powered nature of this study, there are several limitations. First, all patients were studied at 3-Tesla with a single vendor and sequence, and therefore we did not examine the impact of different field strengths or heart rate-independent sequences which may be more robust during tachycardia mediated hyperemia (e.g., shortened MOLLI), which limits the generalizability of our findings [37]. However, these alternative sequences may introduce a loss of precision as a tradeoff, and modifications to the original MOLLI protocol have improved heart rate robustness. Furthermore, given that the mean peak heart rate was less than 100 beats per minute in our population, it is unlikely to have had a significant detrimental effect on the MOLLI sequence [38]. While quantitative CMR perfusion offers greater objectivity and may be more sensitive than qualitative perfusion analysis in determining CAD severity [39], and enables a direct comparison between two quantitative techniques, it remains primarily a research tool with limited model-, vendor-, and field-specific data. Therefore, we compared performance with a qualitative perfusion read to ensure our findings are readily translatable to clinical practice*.* Additionally, the sequence of performing stress T1 mapping and perfusion imaging was not randomized due to the requirement to administer contrast. Finally, we were unable to assess the impact of concomitant coronary microvascular dysfunction (which was not assessed invasively in our study), and may lead to an attenuated stress T1 response in the absence of significant epicardial CAD, but equally may result in false positive stress-perfusion reads.

## 6 Conclusions

Myocardial stress ΔT1 demonstrates poor diagnostic performance for detecting obstructive CAD in patients with suspected angina. This robustly conducted and adequately powered study confirms that stress T1 mapping lacks sufficient clinical utility for the reliable detection of obstructive CAD, underscoring the need to explore alternative contrast-free methods.

## Funding

JRA was supported by a NIHR Clinician Scientist Award (CS-2018–18-ST2–007). GPM was supported by a NIHR Research Professorship (RP-2017–08-ST2–007).

## Author contributions

**Simran Shergill:** Writing – review & editing, Writing – original draft, Formal analysis, Data curation. **Mohamed Elshibly:** Writing – review & editing, Data curation. **Anju Velvet:** Writing – review & editing, Formal analysis. **Aida Moafi:** Writing – review & editing, Formal analysis. **Rachel England:** Writing – review & editing, Resources, Investigation, Formal analysis. **Kelly S. Parke:** Writing – review & editing, Resources, Investigation, Formal analysis. **Joanne V. Wormleighton:** Writing – review & editing, Software, Resources. **David Adlam:** Writing – review & editing, Supervision. **Sandeep S. Hothi:** Writing – review & editing, Supervision. **Peter Kellman:** Writing – review & editing, Supervision, Investigation. **Alasdair McIntosh:** Writing – review & editing, Supervision, Formal analysis. **Alex McConnachie:** Writing – review & editing, Supervision, Funding acquisition, Formal analysis. **Andrew Ladwiniec:** Writing – review & editing, Supervision, Investigation. **Gerry P. McCann:** Writing – review & editing, Supervision, Funding acquisition, Formal analysis. **J. Ranjit Arnold:** Writing – review & editing, Supervision, Methodology, Investigation, Funding acquisition, Formal analysis, Conceptualization.

## Declaration of competing interests

The authors declare the following financial interests/personal relationships which may be considered as potential competing interests: GPM is an editorial board member for JCMR, but had no involvement in the peer review of this article and had no access to information regarding its peer review. Full responsibility for the editorial process for this article was delegated to another journal editor. The remaining declare that they have no known competing financial interests or personal relationships that could have appeared to influence the work reported in this paper.

## Footnotes

Appendix A Supplementary data associated with this article can be found in the online version at doi:10.1016/j.jocmr.2026.102701.
