# Supplementary material for: Stress T1 mapping for the detection of obstructive coronary artery disease: a prospective diagnostic accuracy study
Source: J Cardiovasc Magn Reson. 2026 Jan 29;28(1):102701. doi: 10.1016/j.jocmr.2026.102701 (PMC13237545; doi:10.1016/j.jocmr.2026.102701)
Supplement: Supplementary Table 1 — Supplementary material [file mmc1.docx]

**Supplementary Table 1:** Comparison of resting native T1 values between Siemens 3T scanners

|  | **Skyra, n=15** | **Vida, n=15** | **Mean difference [95% CI]** | **P value** |
| --- | --- | --- | --- | --- |
| Native T1, ms | 1224±30 | 1217±23 | 7 [-13, 27] | 0.470 |

*****Data presented for 30 age (Skyra; 61±4 years vs. Vida; 63±6 years, p=0.308) and sex (females: 7 [47%] vs. 7 [47%]) matched healthy controls. Age and sex adjusted mean difference 9ms [95% CI: -11, 30], p=0.365.

**Supplementary Table 2:** Image quality comparison between rest and stress T1 maps

|  | **Rest T1** | **Stress T1** |
| --- | --- | --- |
| **Excellent** | 80 (69%) | 76 (66%) |
| **Good** | 34 (29%) | 27 (23%) |
| **Moderate** | 1 (1%) | 12 (10%) |
| **Unanalyzable** | 1 (1%) | 1 (1%) |

**Supplementary Table 3:** Native T1 response to adenosine infusion

| **Per patient, n=115** | **Rest T1 (ms)** | **Stress T1 (ms)** | **Mean difference [95% CI]** | **P value** |
| --- | --- | --- | --- | --- |
| Global | 1216.7 ± 48.7 | 1269.9 ± 55.2 | 53.1 [46.1, 60.1] | <0.001 |
| LAD | 1228.5 ± 51.3 | 1282.2 ± 56.4 | 53.7 [46.4, 61.0] |  |
| RCA | 1224.4 ± 42.1 | 1273.2 ± 49.6 | 48.7 [41.6, 55.9] |  |
| LCx^†^ | 1196.0 ± 62.3 | 1251.8 ± 73.3 | 55.8 [46.5, 65.1] |  |
| **Per vessel, n=344** | **Rest T1 (ms)** | **Stress T1 (ms)** | **Mean difference [95% CI]** | **P value** |
| All territories | 1216.4 ± 54.3 | 1269.1 ± 61.7 | 52.7 [48.2, 57.3] | <0.001 |
| No CAD^†^, n=240 | 1219.9 ± 49.1 | 1276.4 ± 56.9 | 56.5 [51.0, 62.1] | <0.001 |
| CAD, n=104 | 1208.3 ± 64.4 | 1252.4 ± 69.0 | 44.0 [36.1, 51.9] | <0.001 |

Data presented as mean ± SD and mean difference [95% confidence interval].

**Abbreviations:** CAD coronary artery disease, CI confidence interval, LAD left anterior descending artery, LCx left circumflex artery, RCA right coronary artery

^†^Rest T1 LCx territory excluded due to artefact: n=1

**Supplementary Table 4:** Comparison of sex-specific T1 response at the vessel level

| **Per vessel** | **Male, n=249** | **Female, n=96** | **P value** |
| --- | --- | --- | --- |
| Rest T1, ms | 1213.3 ± 50.9 | 1224.3 ± 61.9 | 0.093 |
| Stress T1, ms | 1259.9 ± 59.0 | 1292.9 ± 62.1 | <0.001 |
| ΔT1, % | 3.9 ± 3.7 | 5.7 ± 3.1 | <0.001 |

**Supplementary Table 5:** Comparison of the diagnostic performance between stress T1 with stress-perfusion CMR to detect significant CAD, using invasive coronary angiography as the reference standard

|  | **Stress-perfusion CMR** | **Stress T1** | **Mean difference [95% CI]** | **P value** |
| --- | --- | --- | --- | --- |
| **Per vessel, n=345** | ***Reader 1*** |  |  |  |
| Accuracy | 81.2% [77.1%, 84.9%] | 57.4% [52.2%, 62.9%] | 23.8% [17.0%, 30.3%] | <0.001 |
| Sensitivity | 61.5% [51.9%, 71.2%] | 67.3% [57.7%, 76.0%] | -5.8% [-18.3%, 7.0%] | 0.376 |
| Specificity | 89.6% [85.9%, 93.4%] | 53.1% [46.9%, 59.8%] | 36.5% [28.9%, 43.5%] | <0.001 |
|  | ***Reader 2*** |  |  |  |
| Accuracy | 81.7% [77.4%, 85.8%] | 57.4% [52.2%, 62.9%] | 24.3% [17.6%, 30.8%] | <0.001 |
| Sensitivity | 57.7% [48.1%, 66.3%] | 67.3% [57.7%, 76.0%] | -9.6% [-21.7%, 2.9%] | 0.132 |
| Specificity | 92.1% [88.4%, 95.4%] | 53.1% [46.9%, 59.8%] | 39.0% [31.7%, 45.8%] | <0.001 |
| **Per patient, n=115** | ***Consensus*** |  |  |  |
| Accuracy | 83.5% [76.5%, 89.6%] | 58.3% [48.7%, 67.8%] | 25.2% [14.0%, 35.6%] | <0.001 |
| Sensitivity | 76.3% [64.4%, 86.4%] | 78.0% [66.1%, 88.1%] | -1.7% [-15.7%, 12.4%] | 0.808 |
| Specificity | 91.1% [82.1%, 98.2%] | 37.5% [25.0%, 50.0%] | 53.6% [38.4%, 65.3%] | <0.001 |

Proportions expressed as percentage [95% confidence interval].

Two-sided P value for difference

**Abbreviations:** CAD coronary artery disease, CI confidence interval, CMR cardiovascular magnetic resonance

Significant CAD defined by invasive fractional flow reserve (FFR) ≤0.80 in epicardial vessels ≥2mm diameter, or quantitative flow ratio ≤0.80 if FFR not performed

**Supplementary Table 6:** Diagnostic performance of ΔT1 and stress T1 segmental-based approaches and dispersion to detect significant CAD, with invasive coronary angiography as the reference standard

| **Per territory** | **AUC [95% CI]** | **P value** | **Threshold** | **Accuracy** | **Sensitivity** | **Specificity** | **P value*** |
| --- | --- | --- | --- | --- | --- | --- | --- |
| **Lowest segment** | | | | | | |  |
| ΔT1, % | 0.59 [0.52, 0.65] | 0.009 | ≤0.91 | 58.3% | 58.7% | 58.2% | 0.985 |
| Stress T1, ms | 0.59 [0.53, 0.66] | 0.006 | ≤1247.9 | 51.9% | 76.9% | 41.1% | 0.233 |
| **Second lowest segment** | | | | | | |  |
| ΔT1, % | 0.61 [0.54, 0.68] | 0.001 | ≤2.63 | 60.6% | 60.8% | 60.5% | 0.485 |
| Stress T1, ms | 0.64 [0.57, 0.71] | <0.001 | ≤1275.9 | 54.3% | 80.4% | 43.0% | 0.215 |
| **Lowest mean of two adjacent segments** | | | | | | |  |
| ΔT1, % | 0.59 [0.52, 0.65] | 0.008 | ≤2.08 | 56.2% | 66.3% | 51.9% | 0.940 |
| Stress T1, ms | 0.60 [0.54, 0.67] | 0.003 | ≤1273.1 | 49.3% | 83.7% | 34.4% | 0.418 |
| **T1 dispersion (standard deviation)** | | | | | | |  |
| ΔT1, % | 0.52 [0.45, 0.58] | 0.668 | ≤3.34 | 49.0% | 68.6% | 42.5% | 0.036 |
| Stress T1, ms | 0.49 [0.43, 0.56] | 0.855 | ≤25.6 | 57.1% | 34.3% | 68.9% | 0.016 |

*P value for the difference in AUC between given method and territory-based analysis

Significant CAD defined by invasive fractional flow reserve (FFR) ≤0.80 in epicardial vessels ≥2mm diameter, or quantitative flow ratio ≤0.80 if FFR not performed **Abbreviations:** AUC area under the curve, CAD coronary artery disease, CI confidence interval
